# Maximum-Likelihood Model Averaging To Profile Clustering of Site Types across Discrete Linear Sequences

**DOI:** 10.1371/journal.pcbi.1000421

**Published:** 2009-06-26

**Authors:** Zhang Zhang, Jeffrey P. Townsend

**Affiliations:** Department of Ecology and Evolutionary Biology, Yale University, New Haven, Connecticut, United States of America; University of Chicago, United States of America

## Abstract

A major analytical challenge in computational biology is the detection and description of clusters of specified site types, such as polymorphic or substituted sites within DNA or protein sequences. Progress has been stymied by a lack of suitable methods to detect clusters and to estimate the extent of clustering in discrete linear sequences, particularly when there is no *a priori* specification of cluster size or cluster count. Here we derive and demonstrate a maximum likelihood method of hierarchical clustering. Our method incorporates a tripartite divide-and-conquer strategy that models sequence heterogeneity, delineates clusters, and yields a profile of the level of clustering associated with each site. The clustering model may be evaluated via model selection using the Akaike Information Criterion, the corrected Akaike Information Criterion, and the Bayesian Information Criterion. Furthermore, model averaging using weighted model likelihoods may be applied to incorporate model uncertainty into the profile of heterogeneity across sites. We evaluated our method by examining its performance on a number of simulated datasets as well as on empirical polymorphism data from diverse natural alleles of the *Drosophila* alcohol dehydrogenase gene. Our method yielded greater power for the detection of clustered sites across a breadth of parameter ranges, and achieved better accuracy and precision of estimation of clusters, than did the existing empirical cumulative distribution function statistics.

## Author Summary

The invention and application of high-throughput technologies for DNA sequencing have resulted in an increasing abundance of biological sequence data. DNA or protein sequence data are naturally arranged as discrete linear sequences, and one of the fundamental challenges of analysis of sequence data is the description of how those sequences are arranged. Individual sites may be very sequentially heterogeneous or highly clustered into more homogeneous regions. However, progress in addressing this challenge has been hampered by a lack of suitable methods to accurately identify clustering of similar sites when there is no a priori specification of anticipated cluster size or count. Here, we present an algorithm that addresses this challenge, demonstrate its effectiveness with simulated data, and apply it to an example of genetic polymorphism data. Our algorithm requires no a priori knowledge and exhibits greater power than any other unsupervised algorithms. Furthermore, we apply model averaging methodology to overcome the natural and extensive uncertainty in cluster borders, facilitating estimation of a realistic profile of sequence heterogeneity and clustering. These profiles are of broad utility for computational analyses or visualizations of heterogeneity in discrete linear sequences, an enterprise of rapidly increasing importance given the diminishing costs of nucleic acid sequencing.

## Introduction

Analysis of discrete linear sequences has played an increasingly important role in biology. In particular, the detection of heterogeneous regions among sequences can aid in understanding the heterogeneous processes that act upon those regions [1],[2]. Therefore, determining whether specified types or categories of sites, such as polymorphic [3] or substituted sites [4] within DNA or protein sequences, are concentrated in specific regions within DNA or protein sequences has become a key component of these analyses [5]–[8]. For instance, detecting regions that feature heterogeneity in substitutions may provide valuable information on the structure and function of DNAs or proteins [9]–[13].

Several parametric and nonparametric methods have been proposed and historically applied to sequence data. Parametric methods include applications of a Fisher's exact test to tallies of site types between regions, or of a likelihood ratio test to identify heterogeneous regions [14],[15]. Alternatively, several heuristic methods may be applied for this clustering [16]. For example, UPGMA (Unweighted Pair Grouping Method with Arithmetic-mean) or NN (Nearest Neighbor), are hierarchical methods that at each step combine the nearest 2 clusters into one new cluster. Iteration of this step is continued until the number of clusters is one. One of NN's variants, *K*-NN (*K*-Nearest Neighbor), differs in its termination condition, stopping the iteration until the *K* clusters are identified, where *K* needs to be defined in advance. Another heuristic approach, *K*-means, uses a partitioning algorithm to break data into *K* clusters, and also requires the number of clusters *K* as a prior knowledge. When regions of a sequence that are expected to have heterogeneous frequencies of a site type may be specified in advance or the number of clusters to be identified is known *a priori*, these methods have high power to detect clustering [17]. However, they require *a priori* assignment of partitions. When no *a priori* expectation of cluster size or cluster number may be specified, extant studies have usually relied on “sliding window” methods [18]–[23]. For example, Pesole *et al.* (1992) labeled invariable site as ‘1’ and variable site as ‘0’, and applied a sliding window to identify whether ‘1’s are significantly clustered [24]. Pesole *et al.* calculated a heuristic score based on the presence or absence of site types within a window that processes serially across the sequence of interest.

Advantages of sliding window methods include their intuitive conceptual basis and their striking output: an autocorrelated plot of the score that may be superimposed upon the sequence, providing a visual appraisal of the level of clustering at every site. However, sliding window methods have two related major disadvantages [25]. First, they generally offer only crude non-parametric means for statistical significance testing. The autocorrelation of serial scores severely complicates attempts to develop more insightful parametric approaches to sliding window significance testing, making parameter estimation with confidence intervals either challenging or impossible. Second, the need to specify a window size presents a user with a procedural ambiguity. Without a unified statistical framework, there is no strong justification for selection of one window size over another. In such a situation, it may even be tempting to invert the procedure of statistical inference and select a window size that produces an autocorrelated score plot consistent with a particular scientific hypothesis, as opposed to the valid procedure of selecting a window size by an objective statistical optimality criterion.

Because of these disadvantages of the sliding window methods, several nonparametric statistical methods that do not assume prior knowledge have been suggested or implemented to detect clustering in discrete linear sequences. These methods include runs tests [26]–[28] and empirical cumulative distribution function (ECDF) statistics [29],[30]. Runs tests use the “longest unbroken run” between sites of interest as a test statistic for clustering, where a run is defined as consecutive length between events [26]. This test statistic provides very weak power, because it uses very little of the relevant information about the phenomenon of interest, ignoring all runs other than the longest. Statistics based on the longest two runs, longest three runs, or even on a summary of the full distribution of run lengths have been discussed, but remain weak tests. For instance, the variance in distance between site types of interest may be calculated and used as a test statistic for the detection of clusters of sites, where a high variance is indicative of clustering [29]. This test statistic incorporates information about the length of all the runs, but does not capture all of the relevant information: it discards all information about the relative position of runs of different lengths. A sequence with all of its shorter runs in one region would be more clustered than one with short runs distributed evenly.

Currently, the most powerful nonparametric method is the ECDF. It features the cumulative difference between the observed and expected proportion of variant sites to identify regions that differ from other regions in number of substitutions. Under a null model that assumes no heterogeneous region(s) within sequences, this difference remains close to zero. Its significant departure from zero is an indicator for rejecting the null model [29],[30]. Although ECDF has been used to detect heterogeneity in several studies [31]–[35], its power can be affected by the location of the heterogeneous region [30]. Moreover, a parametric method may perform even better across a wide range of datasets.

Most extant methods that have been proposed to detect heterogeneous clusters among sequences suffer from poor power to detect clustering when it is present. The problem is made especially challenging by a tradeoff wherein increasing power to detect clustering also increases overparameterization or false positive rates. Methods that have high power are prone to identify clustering even in random sequences, because even in short sequences, there are so many potential patterns of clustering to evaluate. In this paper, we propose a hierarchical clustering method, model averaged clustering by maximum likelihood (MACML), requiring no priori knowledge of cluster size or cluster count, that provides greater statistical power in detecting heterogeneous regions. MACML adopts a divide-and-conquer approach to hierarchically detect heterogeneous regions and repeat similar analysis for each identified region, unlike most hierarchical methods that do not revisit clusters once they are constructed [17],[36],[37]. To address issues of overparameterization, MACML employs model selection and model averaging techniques that lead to intuitively appealing profiles of sequence heterogeneity and that facilitate description of clustered sites in discrete linear sequences. We describe MACML in detail and provide comparative results in the form of an in-depth evaluation of simulated datasets and an empirical sequence data set on polymorphisms in the *Drosophila* alcohol dehydrogenase gene.

## Materials and Methods

### Algorithm

To apply MACML to locate regional clusters with different specified site types requires a general input sequence *X* with *N* sites, denoted as
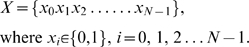
(1)For example, to examine heterogeneity of substitution, an aligned set of homologous sequences is converted into *X*, in which each site is scored entries *x_i_* of 0 representing identity, and 1 representing a variant or variable site [30]. Similarly, a sequence to be analyzed for detection of GC heterogeneity can be converted by setting G/C = 1 and A/T = 0. Notations used to describe our algorithm are summarized in Table 1.

**Table 1:** Notation.

Parameter	Description
*N*	Length of aligned sequences
*X*(*x*_0_ *x*_1_ *…x_N_*_-*1*_)	Sequence, where 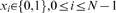
*N*	Number of variant sites, 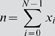
*c_s_*	Start position of cluster
*c_e_*	End position of cluster
*n_s_*	Number of variant sites within the starting region
*n_c_*	Number of variant sites within the cluster region
*n_e_*	Number of variant sites within the ending region
*Q*	Percentage of variant sites within the cluster, 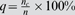
*p*_0_	Variant rate outside of cluster
*p_c_*	Variant rate inside of cluster
*R*	Ratio of variant rates within cluster to outside of cluster, 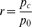
*L*_0_	Maximized likelihood value under the null model
*L_c_*	Maximized likelihood value under the clustering model
*L*	Maximized likelihood value
*K*	Number of parameters
*L*	Sample size
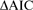	Difference of AIC between the clustering model ( 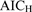 ) and the null model ( 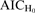 ), 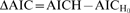
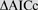	Difference of AICc between the clustering model ( 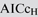 ) and the null model ( 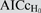 ), 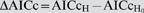
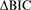	Difference of BIC between the clustering model ( 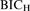 ) and the null model ( 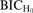 ), 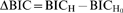

#### Null model

In a sequence with *N* sites, we denote the number of variant sites as 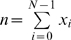. Under a null model, rates of appearance of variants across all sites are the same, equaling 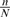. Consequently, the likelihood of the null model is
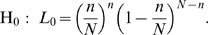
(2)


#### Clustering model

**Figure 1:**
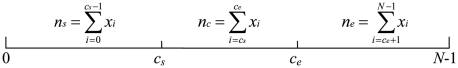
Illustration of parameters for clustering in a sequence. Variables *c_s_* and *c_e_* are the start position and end position of cluster, respectively. Empirical parameters *n_s_*, *n_c_*, and *n_e_* are the number of variant sites in the beginning, central, and ending regions, respectively, such that *n* = *n_s_*+*n_c_*+*n_e_*.

To derive a model incorporating heterogeneity (regional clustering of sites with different variant rates in each region), the entire sequence may first be partitioned into three regions. A central region is bounded by regional endpoints *c_s_* and *c_e_* (0≤*c_s_*<*c_e_*≤*N*-1) (see Figure 1). We may then count the number of variant sites in the starting (*n_s_*), central (*n_c_*), and ending (*n_e_*) regions, respectively. Assuming for the moment that any differential substitution heterogeneity resides in sequence from *c_s_* to *c_e_*, then a likelihood for the clustering model may be formulated as
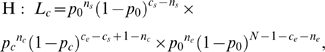
(3)where 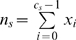, 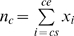, 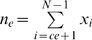, 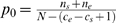, and 
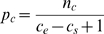
.

Based on these determinate measures associated with the model, we define

- *p*_0_<*p_c_*: The central region (*c_s_*, *c_e_*) is a hot spot, indicating a higher probability of variant sites relative to regions flanking it.
- *p*_0_>*p*_c_: The central region (*c_s_*, *c_e_*) is a cold spot, suggesting a lower probability of variant sites relative to regions flanking it.

Note that if *c_s_* = 0, or if *c_e_* = *N*–1, then there are only two putative regions. The formulation nevertheless applies unchanged.

#### Model selection

Different regional endpoints *c_s_* and *c_e_* lead to a set of diverse, divergently parameterized candidate models (Equation 3) with a range of likelihood values. To decide which model best fits the data and to examine whether a cluster deviates significantly from neighboring sequence, we incorporate several model selection criteria [38]:

- Akaike Information Criterion (AIC) [39]. AIC quantifies the information lost by approximating the true model. AIC incorporates both the maximized likelihood value (*L*) and the number of parameters (*k*). Namely, 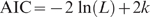. The smaller the AIC, the better the fitness (as in the AICc and BIC below). If the clustering model better fits the data than the null model, then the difference between the cluster model (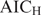) and the null model (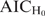) will be large and negative:
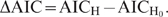
(4)
- Akaike Information Criterion (corrected) (AICc) [40]. A modification of AIC, AICc accounts not only for *L* and *k*, but also for sample size (*l*). 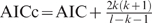. We compare the AICc under the clustering model (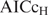) to the AICc under the null model (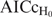). When 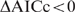, this difference indicates rejection of the null model:
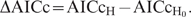
(5)
- Bayesian Information Criterion (BIC) [41]. As in the AICc, BIC is a function of *L*, *k* and *l*, but with a different functional form, where 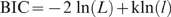. Thus, we test whether the BIC under the clustering model (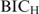) is smaller than that under the null model (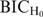), signifying that the clustering model is better than the null model:
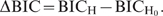
(6)

#### Model averaging

Parameter estimation based on model selection depends upon a single “best” model selected from a set of candidate models [42]. However, because sites may not be variant even when their probability of heterogeneity is high, regional endpoints will rarely be exactly correct. Ideally, the inferred probability of heterogeneity of a site would be influenced in a weighted manner by suboptimal models. To allow all models to contribute to estimation, we make use of model averaging, which accounts for model uncertainty [43]–[45]. To average over models, we assign a weight to each model, and then infer measures of interest across all weighted models. For instance, within the AIC framework, we compute the Akaike weight (*w_i_*, *i* = 1, 2…*m*) for each model,
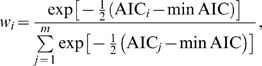
(7)where *m* is the number of models, and minAIC is the smallest AIC value among all models. Measures may then be calculated as the weighted average across all models. Thus, a model-averaged measure of the rate of appearance of a variant at site *i*, *p*(*i*), may be calculated by
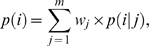
(8)where *p*(*i|j*) is *p*(*i*) given model *j*. Ninety-five percent confidence intervals (C.I.) for the measurement across models may be calculated by sorting all *m* models by their estimated *p*(*i|j*), and sequentially summing the weighted likelihoods of each model from the lowest to the highest values, or from the highest to the lowest values, until the value 0.025 is reached. The *p*(*i|j*) for the last summed model is then the lower or upper C.I., respectively.

#### Implementation

**Figure 2:**
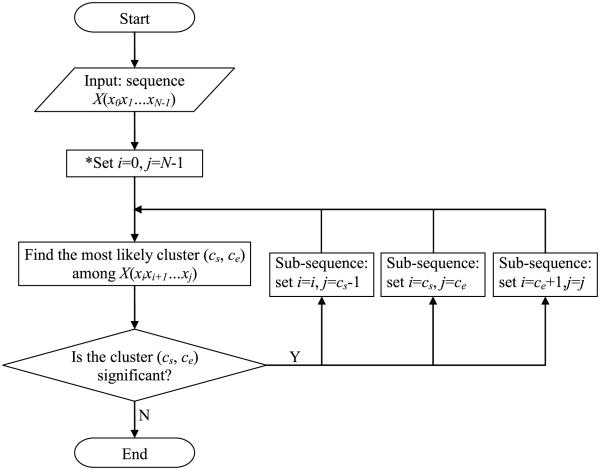
Flowchart for detection of heterogeneous clusters, using the divide-and-conquer approach. *Note that *i* and *j* represent the start position and end position of the sequence or sub-sequence that is currently to be analyzed.

MACML applies a divide-and-conquer approach to hierarchically detect clusters within sequences. After determining the likelihood of all possible models, MACML locates the first cluster, partitions sequences into the three most likely segments, and then repeats a similar analysis for these three segments. The process is iterated on each segment, until all segments and sub-segments of the sequence have failed to demonstrate clustering (see Figure 2).

#### Availability

MACML is written in standard C++ programming language, and its software package, including compiled executables on Linux/Mac/Windows, example data, documentation, and source codes, is freely available for academic use only at http://www.yale.edu/townsend/software.html.

### Simulations

To test the performance of MACML and compare it to the most powerful extant method, ECDF, we simulated sequences for analysis for which the rates of variant sites were known a priori. For each simulated sequence, we randomly generated the start and end positions of the cluster, positions of variant sites within the cluster region, and positions of variant sites within the non-cluster region (see Figure 1). To avoid stochastic errors, we repeated simulations *M* = 10000 times for each parameter combination. Thus, each performance measure was determined from *M* replicates.

#### Power analysis

For each replicate, the expected start position and end position of cluster were denoted as *c_s_* and *c_e_*, respectively. Denoting the corresponding estimated values as 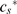 and 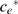, we defined the power to detect clusters within sequences as the proportion of all replicates that satisfies 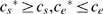 and 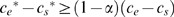, where the permissive zone parameter 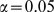. The permissive zone allows each algorithm to just slightly misidentify the start and end of the cluster, improving the scope of the results of our simulations. Without a permissive zone, any algorithm misidentifies the start and end sites of the cluster with such a high frequency that computation becomes burdensome.

#### Accuracy & precision

An alternative assessment criterion, the Kullback-Leibler (KL) divergence [46], requires no permissive zone and provides a more technically satisfactory assessment of the accuracy and precision of the method. The KL divergence calculates how divergent two probability distributions are; in this case, it is used to compare the probabilities of variant sites determined from MACML to probabilities that are known because they were simulated. *M* replicates with *N* sites were simulated for each parameter combination, so that replicates may be indexed by 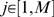 and sites may be indexed by 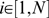. We denote *p_j_*(*i*) and 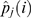 as the expected and estimated values of variant rate at site *i* of replicate *j*, respectively. The KL divergence measures the difference between the two distributions 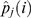 and *p_j_*(*i*), and is defined as
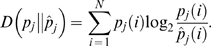
(8)With *M* replicates for each parameter combination, the accuracy may be characterized by the average KL divergence over *M* replicates,
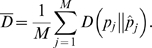
(9)Accordingly, the precision may be calculated as
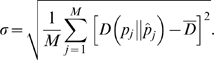
(10)


#### Simulation parameters

The power to detect heterogeneous clusters is a function of the number of variant sites (*n*), the sequence length (*N*), the percentage of variant sites within the cluster (*q*), the ratio (*r* = *p_c_*/*p*_0_) of variant rates within cluster (*p_c_*) to outside of cluster region (*p*_0_), and the number of clusters. We systematically varied parameters of the simulations to obtain a thorough description of algorithm performance.

1. Effects of *n* and *q*. We varied *n* across four values (10, 50, 100 and 200), and *q* from 10% to 90% (and separately *q* = 0% or *q* = 100%, see below), using *r* values 5∶1 for a hot spot and 1∶5 for a cold spot, respectively (consistent with analyses in previous studies [30]). We generated 10000 sequences with *N* = 1000 sites for each parameter combination.
2. Effect of *r*. We set *q* = 60%, *N* = 1000 and *n* = 100. Simulated sequences were generated by varying *r* from 2 to 10 for hot spots, and from 0.1 to 0.9 for cold spots, respectively (10000 replicates for each case). We also examined *r* = 1∶1, implying equal variant rates across the whole sequence. Likewise, *q* = 0% or 100% would indicate that zero or all substitution(s) occur within the central cluster. These extremes represent sequences with entirely randomly located substitutions under the null model. In the context of AIC, AICc or BIC, the power for these sequences represents the error of overparameterization. In the context of ECDF, the power represents the error of the false positive rate. For this reason, sequences under this null model were simulated by using *N* = 1000 and *n* = 10, 50, 100 and 200.
3. Effect of *N*. We fixed *q* = 60% and *n* = 30. Setting *r* = 5∶1 and 1∶5 for hot spots and cold spots, respectively, we generated simulated sequences using values of *N* ranging from 100 to 1000 (10000 replicates for each case).
4. Effect of the number of clusters. To examine the power of detecting multiple clusters among sequences, we took an approach based on that of Tang and Lewontin [30]. One hot spot was set with width 40% of the entire sequence length, then divided into two or more smaller hot spots with equal length, with a cold spot of equal length intervening. We randomly generated not only the start and end positions for the hot spot, but also positions of variant sites for each divided hot spot (this part of our procedure differs moderately from Tang and Lewontin [30], providing a more robust exploration of the power of the methods). Employing four *n* values (10, 50, 100 and 200), we simulated sequences with 1000 sites, with 10000 replicates for each parameter combination.

### Empirical data

We retrieved the *Drosophila* alcohol dehydrogenase (*Adh*) gene within five species of *Drosophila melanogaster* species subgroup (*D. melanogaster*, *D. sechellia*, *D. simulans*, *D. yakuba* and *D. erecta*) from FlyBase [47]. The aligned sequences of *Drosophila Adh* gene can be available at http://www.yale.edu/townsend/datasets.html.

## Results

### Effects of the number of variant sites and the percentage of variant sites within the cluster

**Figure 3:**
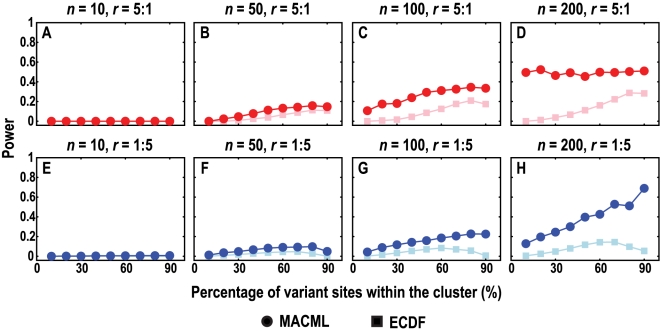
Comparison of the power to detect heterogeneous clusters, evaluating a range of percentages of variant sites within the cluster (*q*). The ratio (*r*) of variant rates within the cluster to outside of the cluster was set to 5∶1 (panels A to D) and 1∶5 (panels E to H), representing hot spots (red) and cold spots (blue), respectively. Four values of *n* were used: 10 in panels A and E, 50 in panels B and F, 100 in panels C and G, and 200 in panels D and H. Each point represents the average of 10000 replicate simulated sequences, with each sequence composed of 1000 sites. The results shown were generated implementing the AIC for model selection. Similar results were obtained implementing the other criteria and incorporating model averaging (see Table S1).

**Figure 4:**
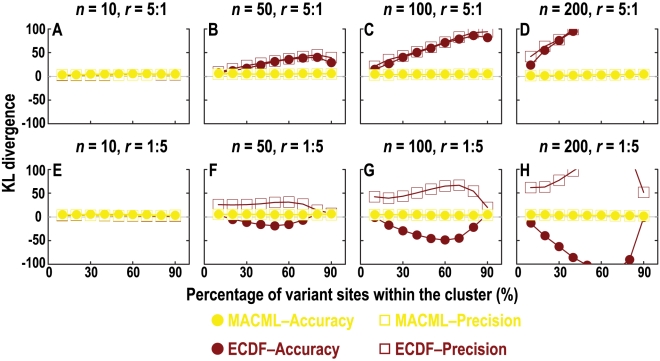
Comparison of accuracy and precision based on the Kullback-Leibler (KL) divergence, evaluating a range of percentages of variant sites within the cluster (*q*). The KL divergence was used as a metric of the distance between the estimated distribution and the expected known distribution. A measure of the KL divergence approaching zero, indicates the two distributions are approaching identity. The ratio (*r*) of variant rates within the cluster to outside of the cluster was set to 5∶1 (panels A to D) and 1∶5 (panels E to H), representing hot spots and cold spots, respectively. Four values of *n* were used: 10 in panels A and E, 50 in panels B and F, 100 in panels C and G, and 200 in panels D and H. Each point represents the average of 10000 replicate simulated sequences, with each sequence composed of 1000 sites. The results shown were generated implementing the AIC for model selection.

The powers of MACML and ECDF were plotted against the percentage of variant sites within the cluster (*q*) under different numbers of variant sites (*n*) in Figure 3 and the corresponding accuracy and precision were plotted in Figure 4. Evaluating the methods based on their power to detect clusters within sequences with different *q* and *n*, MACML outperformed ECDF for nearly all the parameter combinations tested (Figure 3). When *n* was very small, both methods exhibited extremely low power for detecting hot spots (*n* = 10 in Figure 3A). At intermediate values of *n*, MACML and ECDF exhibited increasing power with *q* (Figure 3B and 2C). While ECDF approached the power of MACML when *q* was large, MACML remained more powerful across the full range of *q* (Figure 3B to 2D).

The power of MACML and ECDF to detect cold spots was also low when *n* was small (*n* = 10 in Figure 3E). When *n* increased to 50, the power of MACML and ECDF peaked at intermediate values of *q* (Figure 3F). At higher levels of *n* = 100 (Figure 3G) and *n* = 200 (Figure 3H), ECDF continued to peak at intermediate values of *q*, whereas the power of MACML continued to rise with *q*. Across the parameter ranges examined, MACML consistently exhibited greater power than ECDF.

The accuracy and precision of MACML and ECDF were estimated by the Kullback-Leibler (KL) divergence, which is a measure of the difference between the expected and estimated distributions of variant rates. In assessing the accuracy based on the KL divergence, therefore, there are three potential scenarios: a good match between the estimated and expected variant rates when a KL divergence is near zero, an underestimation of variant rates when KL divergence is positive, and an overestimation of variant rates when KL divergence is negative. The precision based on the KL divergence is also better when it is closer to zero. Unlike the accuracy, precision based on the KL divergence cannot be negative (Equation 12).

Evaluating the accuracy and precision based on the KL divergence, MACML performed better than ECDF for most of the cases examined (Figure 4). The accuracy and precision of MACML and ECDF for detecting hot spots were very good (near zero) when *n* was small (Figure 4A). When *n* became large, MACML exhibited good accuracy and precision, whereas the accuracy and precision of ECDF diverged positively from zero with increasing *q* (Figure 4B to 3D). This divergence was augmented when *n* was extremely large (Figure 4D).

When *n* is small (*n* = 10 in Figure 4E), both MACML and ECDF also exhibited good accuracy and precision for the detection of cold spots. At large values of *n* (Figure 4F to 3H), ECDF exhibited good accuracy and precision only when *q* was smaller (10%) or larger (90%). At intermediate values of *q*, the accuracy of ECDF diverged from the ideal negatively. The precision of ECDF diverged from the ideal as well. This divergence was augmented when *n* was extremely large (*n* = 200 in Figure 4H). In summary, MACML exhibited good accuracy and precision for nearly all tested cases.

### Effect of the ratio of variant rates within cluster to outside of cluster

**Figure 5:**
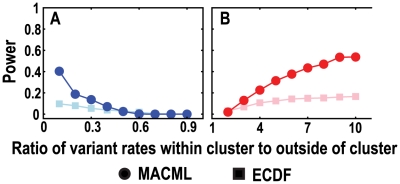
Comparison of the power to detect heterogeneous clusters, evaluating a range of ratios of variant rates within the cluster to outside of the cluster. Cold spots (panel A) and hot spots (panel B) were represented by blue and red, respectively. The percentage of variant sites within the cluster (*q*) was set 60%. Each point represents the average of 10000 replicate simulated sequences, with each sequence composed of 1000 sites. The results shown were generated implementing the AIC for model selection. Similar results were obtained implementing the other criteria and incorporating model averaging (see Table S2).

**Figure 6:**
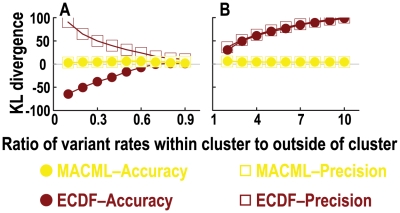
Comparison of accuracy and precision based on the Kullback-Leibler (KL) divergence, evaluating a range of ratios of variant rates within the cluster to outside of the cluster. The KL divergence was used as a metric of the distance between the estimated distribution and the expected known distribution. A measure of the KL divergence approaching zero, indicates the two distributions are approaching identity. Variant sites were simulated with known distributions containing cold spots (panel A) and hot spots (panel B). The percentage of variant sites within the cluster (*q*) was set 60%. Each point represents the average of 10000 replicate simulated sequences, with each sequence composed of 1000 sites. The results shown were generated implementing the AIC for model selection.

The powers of MACML and ECDF were plotted against the ratio of variant rates within cluster to outside of cluster in Figure 5, and the corresponding accuracy and precision were plotted in Figure 6. The difference in power between MACML and ECDF was least remarkable for the detection of cold spots (Figure 5A). At values of the ratio of variant rates within cluster to outside of cluster ranging from 0.3 to 0.9, differences in power between both methods were relatively small, whereas at values of the ratio <0.3, MACML showed much greater power to detect cold spots than did ECDF (Figure 5A). The power of MACML to detect hot spots consistently increased with increasing ratio (Figure 5B). Although the power of ECDF increased with the ratio as well, its power was much lower than the power of MACML across the examined ranges of values of the ratio (Figure 5B).

MACML provided good accuracy and precision (near zero) for detecting cold spots, whereas the accuracy of ECDF diverged negatively and the precision of ECDF diverged from the ideal as well (Figure 6A). This divergence was more notable at values of the ratio <0.7 (Figure 6A). With regard to hot spots, the accuracy and precision of ECDF diverged positively across values of the ratio from 2 to 10 (Figure 6B). As the ratio was increased, this divergence became more remarkable. In contrast, MACML exhibited better accuracy and precision for most of the examined cases (Figure 6B).

According to their definitions, the ratio of variant rates within cluster to outside of cluster = 1∶1, *q* = 0%, or *q* = 100% represent sequences with entirely randomly located substitutions under the null model. Therefore, we compared three criteria adopted by MACML and examined their errors of overparameterizing the clustering model when no clustering was imposed during the sequence generation. MACML and ECDF demonstrated high overparameterization and false positive rates, respectively (Table 2). The overparameterization rate of MACML markedly exceeded the false positive rate of ECDF for *n* = 10, *n* = 100 and *n* = 200. Implementing the AIC and AICc did little to moderate overparameterization, whereas implementing BIC significantly moderated overparameterization. Implementing the BIC did not bring overparameterization down to the false positive rate of ECDF for *n* = 10, 100, and 200, but did limit the overparameterization rate to approximately the false positive rate of ECDF for sequences with *n* = 50.

**Table 2:** False positive rates and overparameterization of the clustering model.

Number of variant sites	ECDF	MACML		
		AIC	AICc	BIC
10	0.0646	0.9957	0.9957	0.2214
50	0.2967	1.0000	1.0000	0.2799
100	0.4906	1.0000	1.0000	0.6753
200	0.3987	1.0000	1.0000	0.5217

Note: Values tabulated are the average over 10000 replicate simulated sequences, each composed of 1000 sites.

### Effect of sequence length

**Figure 7:**
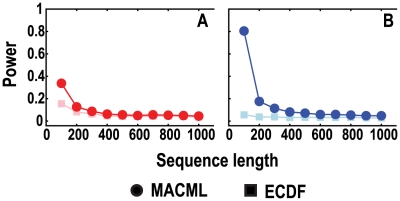
Comparison of the power to detect heterogeneous clusters, evaluating a range of sequence lengths. Ratios of variant rates within the cluster to outside of the cluster were set at 5∶1 (red) and 1∶5 (blue), representing hot spots (panel A) and cold spots (panel B), respectively. Parameters were set at *n* = 30 and *q* = 60%. Each point represents the average of 10000 replicate simulated sequences. The results shown were generated implementing the AIC for model selection. Similar results were obtained implementing the other criteria and incorporating model averaging (see Table S3).

**Figure 8:**
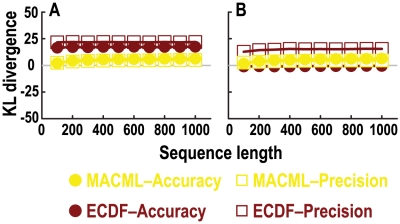
Comparison of accuracy and precision based on the Kullback-Leibler (KL) divergence, evaluating ten sequence lengths. The KL divergence was used as a metric of the distance between the estimated distribution and the expected known distribution. A measure of the KL divergence approaching zero, indicates the two distributions are approaching identity. Ratios of variant rates within the cluster to outside of the cluster were set at 5∶1 and 1∶5, representing hot spots (panel A) and cold spots (panel B), respectively. Parameters were set at *n* = 30 and *q* = 60%. Each point represents the average of 10000 replicate simulated sequences. The results shown were generated implementing the AIC for model selection.

The powers of MACML and ECDF were plotted against sequence length in Figure 7 and the corresponding accuracy and precision were plotted in Figure 8. When sequence length increased from 100 to 1000 sites, MACML and ECDF provided decreasing power to detect both hot spots (Figure 7A) and cold spots (Figure 7B). This decrease was more prominent for MACML than for ECDF. Nonetheless, MACML outperformed ECDF for most of these cases.

The accuracy and precision of MACML and ECDF varied little across all values of sequence length. With increasing sequence length, the accuracy of ECDF diverged from zero positively for hot spots and diverged slightly negatively for cold spots. The precision of ECDF diverged from the ideal positively for both hot spots and cold spots (Figure 8A and 7B). Overall, MACML exhibited better accuracy and precision than ECDF as sequence length increased from 100 to 1000 (Figure 8).

### Effect of the number of clusters

**Figure 9:**
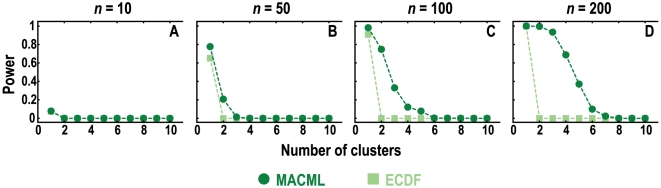
Power to detect multiple heterogeneous clusters. Four *n* values (10 in panel A, 50 in panel B, 100 in panel C, and 200 in panel D) were used for simulations, and each point represents the average of 10000 replicate simulated sequences, with each sequence composed of 1000 sites. The summed width of all clusters was always 40% of entire sequence length. The results shown were generated implementing the AIC for model selection. Similar results were obtained implementing the other criteria and incorporating model averaging (see Table S4).

The powers of MACML and ECDF were plotted against the number of clusters in Figure 9. Under the parameters examined for multiple clusters (see Materials and Methods), MACML and ECDF performed similarly when the sequence had only one cluster to be detected. However, when the number of clusters ranged from 2 to 10, ECDF was unable to detect more than one cluster, whereas MACML had significant power to detect multiple clusters, especially for large values of *n*. In general, the power of MACML was limited for small values of *n* = 10 (Figure 9A) and *n* = 50 (Figure 9B), but much greater for large values of *n* = 100 (Figure 9C) and *n* = 200 (Figure 9D).

### Applied example

**Figure 10:**
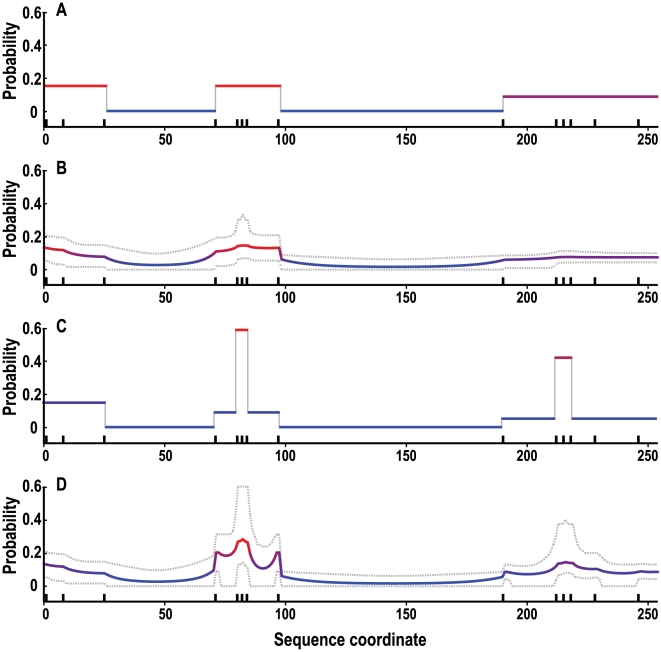
Profile of clustering of polymorphic sites within the *Adh* protein (254 amino acids) in *D. melanogaster*. (A) BIC with model selection, (B) BIC with model averaging, (C) AIC with model selection, and (D) AIC with model averaging (AICc obtained results similar to AIC; data not shown). Colors of sites were based on their estimated probability of polymorphism. A higher percentage of blue indicates low probability of polymorphism, whereas a higher percentage of red indicates larger probability of polymorphism. Polymorphisms are present at sites 2, 9, 26, 72, 81, 83, 85, 98, 191, 213, 216, 219, 229, and 247, depicted by tick marks above the x-axis. Grey lines in panels B and D are composed of the 95% confidence intervals across models for the measured probability for each site.

We applied MACML to detect heterogeneous clusters of polymorphisms within the *Drosophila Adh* gene and to profile potential for polymorphism for each site based on model selection and model averaging, respectively. Identified clusters as well as profiles of the potential for polymorphism were plotted against sequence coordinate (Figure 10). As expected, profiles of potential for polymorphism based on model selection (Figure 10A and 9C) are highly discrete, whereas smoother, continuous profiles are produced based on model averaging (Figure 10B and 9D). When using BIC, MACML detected two clusters along the *Adh* gene and both are cold spots residing between sites 98 and 189 and between sites 26 and 70 (Figure 10A and 9B). In addition to these two cold spots, when using AIC or AICc, MACML also identified two hot spots between sites 80 and 84 and between sites 212 and 218 (Figure 10C and 9D). In contrast, ECDF detected only one cold spot between sites 98 and 211 (data not shown), consistent with previous applications of the method [29],[30].

Detailed clustering results for the *Adh* gene are summarized in Table 3. For the AIC or AICc, the four detected clusters all deviate significantly from the null model (ΔAIC<0 and ΔAICc<0 in Table 3). When sample size is large, like sequence from sites 0 to 253, the ΔAICc asymptotically approaches ΔAIC, and thus their values are nearly same. However, for a smaller sample size, for example, when detecting sub-sequence from sites 71 to 97, ΔAICc is much larger than ΔAIC. By contrast, BIC incorporates a heavier penalty than AIC or AICc and ΔBIC>0 indicated no significant cluster among sub-sequences from sites 71 to 97 or from 190 to 253, whereas AIC and AICc identified two clusters along these two sub-sequences.

**Table 3:** Detailed quantitative analysis of clustering of polymorphism across the *Adh* gene.

Location	*c_s_*	*c_e_*	*p*_0_	*p_c_*	ln*L*_0_	ln*L_c_*	ln*L_c_*–ln*L*_0_	ΔAIC	ΔAICc	ΔBIC
0 ∼ 253	98	189	0.09	0.00	−54.18	−47.65	6.53	−9.05	−9.01	−1.98
0 ∼ 97	26	70	0.15	0.00	−27.71	−22.49	5.22	−6.44	−6.31	−1.27
71 ∼ 97	80	84	0.09	0.60	−12.94	−10.07	2.87	−1.74	−1.24	0.85
190 ∼ 253	212	218	0.05	0.43	−19.91	−16.53	3.38	−2.76	−2.56	1.56

## Discussion

### Comparative analysis of simulated results

The power to detect heterogeneous clustered sites within sequences depended in moderately complex ways on the parameters we examined in this report. Consistent with expectations, our results show that the power of MACML to detect hot spots and cold spots increased with increasing percentage of variant sites within the cluster (Figure 3). Across simulations comparing different percentages of variant sites within the cluster, MACML exhibited both high accuracy and high precision: the estimated variant rates within and outside clusters were close to the expected ones across all parameter combinations (Figure 4). In contrast to MACML, ECDF performed more variably across different percentages of variant sites within the cluster. This inconsistency of performance agrees well with our theoretical analysis on ECDF (Text S1) as well as with results from a previous study [30]. The hot spots and cold spots estimated by ECDF tend to be narrower than the simulated hot spots and cold spots [30]. The misattributed region between the boundary of the estimated hot or cold spot and the corresponding boundary of the simulated hot or cold spot generally gives rise to much greater KL divergence than any other region of the sequence. Thus, the KL divergence of the full sequence tends to be dominated in direction and magnitude by the KL divergence of the region between these boundaries, a region that is usually present as a consequence of the bias in estimation of the width of hot and cold spots. Accordingly, positive divergence from perfect accuracy and precision for hot spots (Figure 4A to 3D) follows from underestimation of the variant rate of this region. Likewise, negative divergence from perfect accuracy and positive divergence from perfect precision for cold spot (Figure 4E to 3H) follows from overestimation of the variant rate of this region.

Across a range of ratios of variant rates within the cluster to outside of the cluster, MACML and ECDF exhibit similar trends in power, but different trends in accuracy and precision. With both methods, a significant difference between variant rates within the cluster and outside of the cluster leads to greater power, and nearly equal rates for all sites results in lower power (Figure 5). The KL divergence measure of the accuracy of ECDF is negative for cold spots and positive for hot spots, respectively (Figure 6). When the variant rate inside of the cluster approaches the variant rate outside of the cluster, estimated and actual variant rates are very close for any cluster model. Therefore, the accuracy and precision of ECDF approach those of MACML, consistent with simulation results (Figure 6). In contrast, as variant rates within the cluster diverge from rates outside the cluster, MACML produces incrementally better accuracy and precision across all parameter combinations (Figure 6).

Both MACML and ECDF exhibit decreasing power with increasing sequence length (Figure 7), presumably as a consequence of the decreasing proportion of variant sites relative to sequence length. Increasing sequence length with a fixed number of variant sites is equivalent to decreasing the number of variant sites with a fixed sequence length. Therefore, it is consistent that the power decreases with decreasing variant sites in Figure 3. This relationship between variant sites and power also agrees well with the results observed when varying the number of clusters (Figure 9), with the additional note that ECDF fails to detect more than one cluster. It is notable that simulations performed by Tang and Lewontin [30] were less general in scope than ours. That is, in Tang and Lewontin [30], the heterogeneous cluster was always centered and the two regions flanking the cluster were always equal in length. As noted by Tang and Lewontin, the power of ECDF is affected when the cluster moves off center [30]. In our simulations, the starting position and ending position of cluster are randomly generated, leading to a random location of the cluster and thus to an unequal length of the two flanking regions (see details in Materials and Methods). For these reasons, our simulations that incorporated random positions of clusters yielded different results in terms of success detecting multiple clusters than were yielded by the simulations of Tang and Lewontin [30].

False positive rates and overparameterization for clustering models were high, as expected as a consequence of the large number of potential cluster boundary sets that are possible. Powerful methods for this class of problem are expected to display high false positive rates, a tradeoff that is natural in statistical inference. Although ECDF presents lower false positive rates, MACML achieves more power than ECDF to reject the null hypothesis when it is not true (Figures 3, 4 and 6). Moreover, MACML achieves markedly greater accuracy and precision of variant rates as determined by the KL divergence (Figures 3, 5 and 7), demonstrating the marked superiority of MACML in selecting the best model of variant rates across a discrete linear sequence. Furthermore, MACML is more capable of detecting multiple clusters among sequences, as demonstrated by simulation (Figure 9) and by application to the empirical data (Figure 10).

### Differences of the adopted criteria

Unlike ECDF, which is not integrated into a model selection framework, MACML adopts AIC, AICc and BIC for model selection. To clarify the differences observed implementing these diverse criteria, the different penalties for additional parameterization that they entail may be compared. Based on the clustering model, two parameters (*c_s_* and *c_e_*) are evaluated (from which *p*_0_ and *p_c_* can be calculated). Therefore, the number of parameters under the clustering model is two, whereas the number under the null model is zero. From equations 4–6, then,
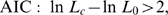
(11)

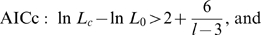
(12)

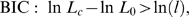
(13)where *l* is sample size, that is, (sub-)sequence length.

**Figure 11:**
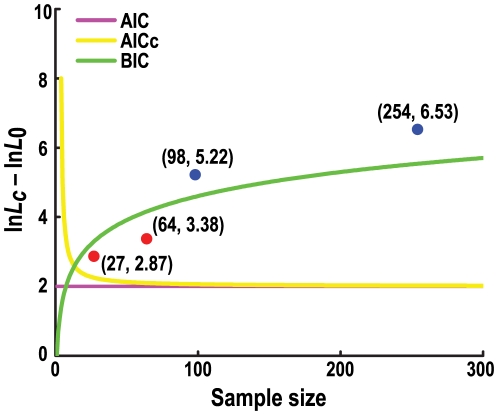
Depiction of the relationships between model selection criteria, ln*L_c_*–ln*L*_0_, and sample size.

The values of ln*L_c_*–ln*L*_0_ may be plotted against sample size (Equations 11–13, Figure 11). AIC yields constant penalties for all values of sample size. For smaller sample size, AICc yields larger penalties than AIC or BIC. When sample size increases to large numbers, the penalty of AICc approaches AIC, and BIC produces much larger penalties than AICc.

For a given value of ln*L_c_*–ln*L*_0_, the three criteria are most likely to give different results with regard to rejection of the null model. The three lines plotted corresponding to the three different criteria in Figure 11 may be helpfully related to the results of our application of MACML to the *Adh* gene. MACML started by detecting a cluster from site 0 to 253. The sample size was 254, and the corresponding value of ln*L_c_*–ln*L*_0_ was 6.53 (Table 3). This cluster is represented by a point (254, 6.53), located above all three lines. This location signifies that the three criteria all reject the null model. After locating the first cluster, MACML proceeded to detect clusters along sub-sequences from 0 to 97, from 98 to 189, and from 190 to 253, until all possible sub-sequences had been tested. As a consequence, it identified several clusters. Two of them are located above the three lines, signifying that all three criteria reject the null model. The remaining two points are located below the BIC line and above the other lines, signifying that BIC does not reject the null model, but that the rest do (Figure 11). This graphical analysis clarifies results in which BIC identified only two cold spots, whereas the other criteria identified an additional two hot spots (Figure 11 and Table 3).

### Significance of profiling heterogeneity

The *Drosophila Adh* is the most studied enzyme that catalyzes the oxidation of alcohols to aldehydes/ketones [48]. It has been extensive reported that several functionally important residues reside in the *Adh* gene: tyrosine-152, lysine-156 and serine-139 are conserved in homologous dehydrogenases and have important roles in catalysis [49]–[53]; glycine-130, glycine-133 and glycine-184 contribute substantially to the structure of the active form [50]; and aspartic acid-64 lies within a coenzyme-binding domain [51]. As shown in Figure 10 and Table 3, these residues were all clustered into the cold spots by MACML, indicating not only their functional conservation and relevance, but also the extent of the region of near-neighbor amino acids that are also conserved. Near-neighbors may be conserved due to their structural and biochemical effects on the known function of these residues. In addition, according to its gene structure, two introns in the *Adh* gene reside between the nucleotide sequences coding for residues 32 and 33 and between the nucleotide sequences coding for residues 167 and 168 [54],[55]. Therefore, the two cold spots identified by MACML extending from residues 26 to 70 and from residues 98 to 189 indicate conservation around the introns.

Heterogeneity of variant rates among specified site types is thought to commonly occur [56]–[59] and may derive from many sources, including functional constraint, gene structure, 3D protein structure, composition bias, mutation bias or recombination [1], [18], [34], [60]–[62]. As indicated by our results based on the simulated data and real data, MACML, equipped with model selection and model averaging, features smooth and continuous profiles of variant rates for each site, and is more accurate and more informative for the detection of multiple clusters among sequences. Therefore, MACML furnishes broad utility for any computational analyses of heterogeneous discrete linear sequences and provides valuable information to aid for a better understanding of the structure and function of DNAs or proteins.

In addition, MACML can be applied to a broad range of applications. For example, MACML would be appropriate for determining whether components of any multicomponent polymer have a clustered structure [33],[63]. It can also be used to detect compositional heterogeneity within sequences [64]–[66] (e.g., heterogeneous GC content by setting G/C = 1 and A/T = 0). Moreover, MACML may provide a framework upon which future modeling of the substitution process may be overlain, assessing heterogeneity in selective pressure acting on different coding sequence regions [60], [67]–[70] and detecting fast-evolving regions in noncoding sequences [71],[72].

### Conclusion

Here we have presented a method, MACML, to detect clustering of a site type in discrete linear sequences. MACML features maximum likelihood estimation, model selection criteria (AIC, AICc, and BIC) and model averaging to profile sequence heterogeneity. It employs a divide-and-conquer approach to hierarchically detect multiple clusters within sequences, without requiring a priori knowledge for cluster size or number. We compared MACML with the most powerful competing method, the ECDF, by exploring a full range of parameter space using computer simulations, and by performing an analysis of empirical data. Our comparative results show that across a wide range of parameter combinations, MACML outperforms ECDF not only by exhibiting greater power to detecting hot spots and cold spots. Thus, it represents a powerful exploratory tool for profiling clustering in discrete linear sequences. Although discoveries using MACML should be considered tentative, it yields greater resolution than any other method, providing a significant advance for the analysis of clustering of sites within discrete linear sequences.

## Supporting Information

Table S1 Power to detect heterogeneous clusters, evaluating a range of percentages of variant sites within the cluster (*q*)
(0.02 MB XLS)

Table S2 Power to detect heterogeneous clusters, evaluating a range of ratios of variant rates within the cluster to outside of the cluster (*r*)
(0.02 MB XLS)

Table S3 Power to detect heterogeneous clusters, evaluating a range of sequence lengths
(0.02 MB XLS)

Table S4 Power to detect multiple heterogeneous clusters
(0.02 MB XLS)

Text S1 Analysis on ECDF
(0.05 MB DOC)
