# Supplementary material for: Maximum-Likelihood Model Averaging To Profile Clustering of Site Types across Discrete Linear Sequences
Source: PLoS Comput Biol. 2009 Jun 26;5(6):e1000421. doi: 10.1371/journal.pcbi.1000421 (PMC2695770; doi:10.1371/journal.pcbi.1000421)
Supplement: Text S1 — Analysis on ECDF (0.05 MB DOC) [file pcbi.1000421.s005.doc]

Supporting information

# Maximum-likelihood model averaging to profile clustering of site types across discrete linear sequences

Zhang Zhang and Jeffrey P. Townsend*

Department of Ecology and Evolutionary Biology, Yale University, New Haven, Connecticut 06520, United States of America

*Corresponding author

Suppose that *n* is the number of variant sites, *N* is the length of sequences, *w* is the width of cluster, *q* is the percentage of variant sites within the cluster, and *r* is the ratio of variant rates within cluster (*p_c_*) to outside of cluster (*p*_0_). Hence, and . As a result, *w* can be formulated as a function of *q* and *r*,

$w=\frac{1}{1+\left(\frac{1}{q}-1\right)r}$. (S1)

Therefore, *r*, an indicator of hot spots when *r* > 1 or cold spots when *r* < 1, correlates negatively with *w*, and *q* correlates positively with *w*.

Empirical cumulative distribution function (ECDF) statistics evaluates the cumulative difference *G*(*i*) at site *i* between the observed and expected proportion of variant sites [1]. Under the null model, this difference, *G*(*i*), is zero or nearly zero. A significant departure from zero is an indicator for rejecting the null model, where *x_i_* = 1, *i* = 0, 1, 2…*N*–1 (for details, see [1]). In our simulations, the cluster is bounded by the starting site (*c_s_*) and the ending site (*c_e_*). Thus, the cumulative difference for sites *c_s_* and *c_e_* is

and , (S2)

respectively, where *n_s_* is the number of variant sites from site 0 to *c_s_*−1, and *n_c_* is the number of variant sites from *c_s_* to *c_e_*, that is, *n_c_* = *nq*.

Based on equation S1 and S2, the difference between *G*(*c_e_*) and *G*(*c_s_*) can be formulated as

. (S3)

It can be seen according to equation S1 and S2 that

- For small *q*, $\lim_{q\to0} \Delta G=\lim_{q\to0} (q-w)=\lim_{q\to0} q-\lim_{q\to0} w=0$, suggesting low power.
- As *q* increases, *w* accordingly becomes large, leading to $\Delta G\neq0$, indicating an increase in power.
- For extremely large values of *q*, *w* correspondingly approaches 1, that is, $\lim_{q\to1} w=1$. Hence, $\lim_{q\to1} \Delta G=\lim_{q\to1} (q-w)=\lim_{q\to1} q-\lim_{q\to1} w=0$, again indicating low power. This asymptote is approached especially rapidly for cold spots, because *w* also depends on *r*: the smaller *r*, the closer *w* approaches 1 (Equation S1).

Reference:

1. Tang H, Lewontin RC (1999) Locating regions of differential variability in DNA and protein sequences. Genetics 153: 485-495.
